# Aging, cardiac repair and Smad3

**DOI:** 10.18632/aging.101567

**Published:** 2018-09-20

**Authors:** Bijun Chen, Shuaibo Huang, Nikolaos G Frangogiannis

**Affiliations:** 1 The Wilf Family Cardiovascular Research Institute, Department of Medicine (Cardiology), Albert Einstein College of Medicine, Bronx, NY 10461, USA

**Keywords:** Transforming Growth Factor-beta, myocardial infarction, aging, fibroblast, heart failure, extracellular matrix

The adult mammalian myocardium lacks endogenous regenerative capacity; thus, following myocardial infarction, the heart heals through formation of a collagen-based scar. Cardiac repair is dependent on a superbly orchestrated inflammatory cascade that sequentially recruits inflammatory cells, fibroblasts and vascular cells in the infarct zone. Expansion of fibroblast populations in the infarcted myocardium and conversion into activated myofibroblasts play a critical role in infarct healing, maintaining the structural integrity of the ventricle and preventing cardiac rupture. However, exaggerated myofibroblast activation may promote excessive deposition of extracellular matrix proteins in the infarct border zone and in the viable remodeling myocardium, precipitating heart failure [1]. Members of the Transforming Growth Factor (TGF)-β superfamily have been implicated in activation of fibroblasts in healing and remodeling tissues. TGF-βs act by transducing signaling cascades mediated through a series of intracellular effectors the Smads, or through Smad-independent pathways. Smad2/3 signaling is activated in all cell types involved in cardiac repair [2,3], and may modulate inflammatory, reparative and remodeling responses.

## Cell-specific effects of Smad3 signaling in the infarcted myocardium

In a recently published study, we generated cell-specific Smad3 knockout mice, in order to investigate the role of Smad3 signaling in regulating fibroblast and cardiomyocyte function following myocardial infarction [4]. We found that cardiomyocyte Smad3 signaling has no effects on cardiac homeostasis, but promotes cardiomyocyte apoptosis and accentuates dilative remodeling, enhancing matrix metalloproteinase expression, and increasing nitrosative stress following myocardial infarction. In contrast, Smad3 signaling in activated infarct myofibroblasts is protective, restraining fibroblast proliferation and contributing to scar organization by stimulating integrin-dependent interactions between the fibroblasts and the extracellular matrix In the infarcted myocardium, myofibroblasts are organized in arrays, exhibiting alignment along the direction of the ventricular wall. Myofibroblast-specific loss of Smad3 perturbs alignment of myofibroblast arrays in the infarct, leading to formation of a disorganized scar. Disturbances in scar formation in myofibroblast-specific Smad3 knockout mice are associated with an increased incidence of cardiac rupture and with accentuated dilative remodeling. These findings highlight the crucial reparative role of activated myofibroblasts following myocardial infarction. Moreover, our observations may have major implications in understanding the basis for worse outcome and accentuated post-infarction remodeling in senescent subjects.

## The role of TGF-β/Smad3 signaling in repair of the senescent heart

Senescent hearts exhibit a modest baseline expansion of the cardiac interstitium, associated with increased collagen deposition, and worse diastolic function [5]. On the other hand, older subjects exhibit impaired reparative responses with a major impact on their prognosis following myocardial injury. Elderly patients have an increased incidence of post-infarction heart failure and accentuated adverse remodeling that cannot be explained by larger infarcts. Using a mouse model of reperfused myocardial infarction, we have previously demonstrated that senescent animals (>24 months of age) exhibit worse adverse remodeling following myocardial infarction, when compared with young mice (3-4 months of age). Age-related adverse post-infarction remodeling is associated with a delayed reparative response, and with markedly reduced collagen deposition in the scar. In vitro, cardiac fibroblasts isolated from senescent hearts have impaired responses to TGF-β stimulation, exhibiting attenuated activation of Smad-dependent signaling [6]. Our recent findings on the crucial reparative role of the Smad3 pathway in cardiac fibroblasts [4], suggest that defective repair in senescent mice may be due, at least in part, to perturbed activation of TGF-β/Smad signaling.

## What is the basis for attenuated reparative Smad-dependent responses in senescent fibroblasts?

**Figure 1:**
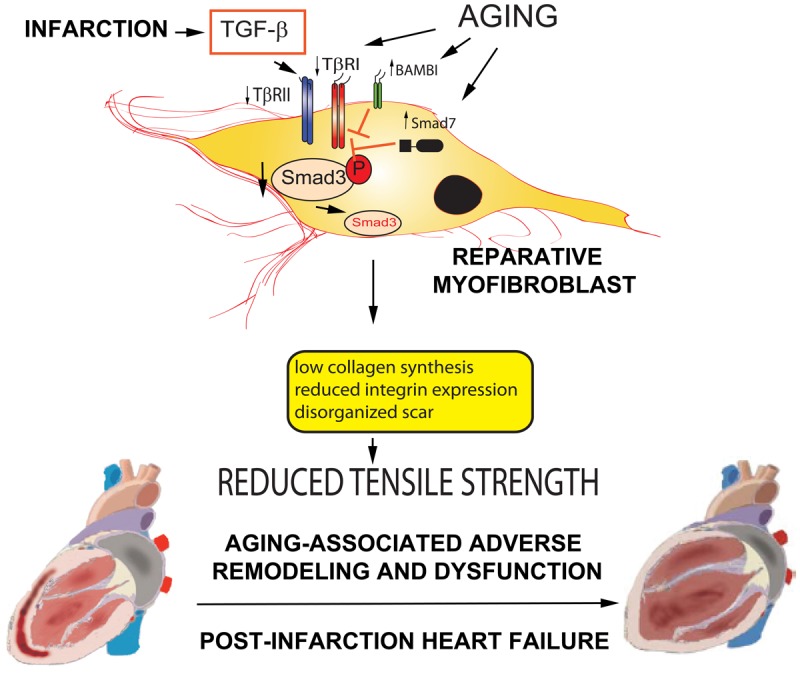
**Age-related accentuation of adverse post-infarction remodeling may be due to an impaired response of reparative fibroblasts to TGF-β.** Our observations suggest that: a) myofibroblast-specific loss of Smad3 disrupts scar organization, increasing adverse remodeling following myocardial infarction, b) senescent hearts exhibit impaired infarct healing, accompanied by decreased collagen deposition, and c) fibroblasts harvested from senescent hearts exhibit blunted Smad2/3 activation in response to TGF-β stimulation. Taken together these observations suggest the intriguing hypothesis that age-associated impairment in TGF-β/Smad signaling in reparative fibroblasts may reduce tensile strength of the healing scar, accentuating adverse remodeling following infarction and causing heart failure. Several mechanisms may account for reduced TGF−β responses in senescent cardiac fibroblasts. Age-associated alterations of the expression of signaling TGF-β receptors (TβRI and TβRII) and pseudoreceptors (such as BAMBI) may modulate the response to TGF-β. Activation of phosphatases or induction of inhibitory Smad7, such as Smad7 may attenuate Smad2/3 activation in response to TGF-β. Because aging is associated with baseline activation of a fibrogenic program (that may involve low level increase in basal TGF-β activity), TGF-β-driven induction of endogenous signals that inhibit Smad-dependent signaling may be responsible for blunted responses to the sudden burst in TGF-β activity observed following cardiac injury.

Several distinct mechanisms may explain the perturbed reparative response of senescent cardiac fibroblasts to TGF-β (Figure 1). First, aging may be associated with marked changes in the cellular composition of the cardiac interstitium, leading to selective expansion of cells with low responsiveness to growth factors [7]. Published evidence suggests that defective responses of senescent cardiac fibroblasts are not limited to TGF-β, but may also involve other activating mediators, such as angiotensin II [8]. Second, fibroblasts in senescent hearts may exhibit lower levels of TGF-β receptors, or activation of pseudoreceptors such as BAMBI (BMP and activin membrane-bound inhibitor) that silence TGF-β signaling. Third, senescent fibroblasts may exhibit activation of phosphatases that dephosphorylate Smads, or TGF-β-driven induction of inhibitory Smads (such as Smad7), that inhibit TGF-β signaling responses. The chronic low-level activation of the TGF-β system in senescent hearts may induce baseline expression of endogenous inhibitors of the Smad cascade that attenuate Smad2/3 stimulation in response to acute injury.

## Increasing the reparative reserve of the senescent heart

Taken together, our studies suggest that adverse outcome in senescent subjects surviving myocardial infarction may involve defective TGF-β/Smad3-dependent fibroblast activation. Blunted responses of senescent fibroblasts to growth factors may result in formation of a disorganized scar following infarction, reducing tensile strength and accentuating adverse remodeling and systolic dysfunction (Figure 1). This intriguing hypothesis has not yet been tested in vivo. However, if true, this mechanism of age-associated dysfunction may suggest new strategies to improve outcome following cardiac injury in elderly subjects, by implementing strategies that enhance fibroblast activity and promote repair. Brief and cautious administration of growth factors, along with the injection of biomaterials, or cell therapy with healthy reparative fibroblasts may represent effective new strategies for prevention of post-infarction heart failure in elderly patients surviving myocardial infarction.
